# Identification of pain categories associated with change in pain in patients receiving placebo: data from two phase 3 randomized clinical trials in symptomatic knee osteoarthritis

**DOI:** 10.1186/s12891-018-1938-5

**Published:** 2018-01-17

**Authors:** Asger Reinstrup Bihlet, Inger Byrjalsen, Anne-Christine Bay-Jensen, Jeppe Ragnar Andersen, Claus Christiansen, Bente Juel Riis, Ivo Valter, Morten A. Karsdal, Marc C. Hochberg

**Affiliations:** 1 Nordic Bioscience, Herlev Hovedgade 207, DK2730 Herlev, Denmark; 2 Bioclinica, Tallinn, Estonia; 3 University of Maryland School of Medicine, Baltimore, USA

**Keywords:** Pain categories, Placebo-response, Radiographic progression

## Abstract

**Background:**

Pain is the principal clinical symptom of osteoarthritis (OA), and development of safe and effective analgesics for OA pain is needed. Drug development of new analgesics for OA pain is impaired by substantial change in pain in patients receiving placebo, and more data describing clinical characteristics and pain categories particularly associated with this phenomenon is needed.

The purpose of this post-hoc analysis was to investigate clinical characteristics and pain categories and their association with radiographic progression and placebo pain reduction (PPR) in OA patients as measured the Western Ontario and McMasters Arthritis (WOMAC).

**Methods:**

Pooled data from the placebo groups of two phase III randomized clinical trials in patients with knee OA followed for 2 years were analyzed. Differences between individual sub-scores and pain categories of weight-bearing and non-weight bearing pain over time were assessed. Selected patient baseline characteristics were assessed for association with PPR. Association between pain categories and radiographic progression was analyzed.

**Results:**

The reduction of pain in placebo-treated patients was significantly higher in the composite of questions related to weight-bearing pain compared to non-weight-bearing pain of the target knee. Baseline BMI, age and JSW were not associated with pain change. Pain reduction was higher in the Target knee, compared to the Non-Target knee at all corresponding time-points. A very weak correlation was found between weight-bearing pain and progression in the non-target knee.

**Conclusions:**

These results indicate that the reduction in pain in patients treated with placebo is significantly different between pain categories, as weight-bearing pain was significantly more reduced compared to non-weight-bearing pain. Further research in pain categories in OA is warranted.

**Trial Registration:**

NCT00486434 (trial 1) and NCT00704847 (trial 2)

## Background

Osteoarthritis (OA) is the most common arthritic disease, affecting more than 250 million people in the world [1]. Pain is the principal clinical symptom of osteoarthritis (OA), and while several approved medical treatments for OA pain exist, new safe and effective analgesics are still needed [2]. OA pain itself is considered to be the result of several pathological conditions, manifested as allodynia, hyperalgesia and central sensitization, to varying degrees [3–5]. This diffuse construct of features combined with elements of psychosocial factors has proven particularly difficult to treat [6, 7]. Whether and how knee pain is linked to increased risk of radiographic progression is controversial [8–10]. Indeed OA pain is considered to be multifactorial [3, 4], and there is no clear-cut association of single-factors with disease activity and structural degradation. Clinical research evaluating new efficacious analgesic treatments in OA is also impaired by a substantial placebo-response, requiring large sample sizes, complex study designs or cumbersome methods of study participant selection to facilitate demonstration of statistically significant improvement in reported pain levels [11]. Several mechanisms are thought to be involved in placebo-response [12–14], but data describing if different categories of pain are similarly affected by placebo-response are lacking, and links between pain categories and disease activity remains unclear.

Specialized and validated methods of subtyping pain into elements of central sensitization [15], neuropathic pain [16], among others, exist. The use of more crude, exploratory methods include the subscores of the well-recognized Western Ontario and McMaster Universities Osteoarthritis Index (WOMAC) [17] as a potential indicator of differences in pain categories. In a report by Lo et al. from 2009, the authors found that WOMAC pain while the joint is load-bearing is likely to be linked to bone marrow lesions and effusion assessed using magnetic resonance imaging (MRI), but pain while idle is not, indicating that pain is multifactorial [18]. Further in support of differences behind weight-bearing and non-weight-bearing pain is a report by Hensor et al. analyzing periodic individual WOMAC questions from the OsteoArthritis Initiative (OAI) of people at high risk of developing OA over a period of 7 years, showed that pain while walking up or down stairs was the first to manifest as a symptom of OA, and pain while in bed at night was registered as the last [19].

A report by Stratford et al. further concluded that these two pain categories, pain while weight-bearing vs. non-weight-bearing pain, reflect different pain constructs [20]. Thus, attempts to quantify pain using the total WOMAC pain composite may not be ideal, and additional, exploratory information which may point to differences in pain etiology may be derived from analyzing the two pain constructs separately.

In the absence of existing research describing the origin of pain categories and their association with placebo pain reduction (PPR), this report will assess associations between age, clinical and radiographic features and pain categories with reduction in pain as reported by patients receiving placebo as well as associations between pain categories and risk of radiographic progression in two pooled randomized controlled trials.

## Methods

### Study population

This is a post-hoc analysis of the placebo groups in two, double-blinded, randomized, placebo-controlled and multicenter phase III clinical trials assessing the efficacy and safety of an oral formulation of 0.8 mg salmon calcitonin in patients with painful knee OA (NCT00486434 (trial 1) and NCT00704847 (trial 2)). Each independent trial recruited patients aged 51–80 years with painful OA in the target knee, defined as a Visual Analogue Score of ≥150 mm on the WOMAC pain subscale (500 mm being the maximum score). In study 2, patients scoring ≤150 mm on the pain sub-score were allowed to participate if they also scored ≥510 mm on the WOMAC function sub-scale (1700 mm being the maximum score). The radiographic inclusion criteria for target knees included Kellgren-Lawrence (KL) grades 2 or 3, and a Joint Space Width (JSW) of ≥2.0 mm. A total of 2206 patients were recruited at 19 sites in 11 countries. Details regarding trial design and results are published elsewhere [21]. The present analysis includes the 771 placebo-treated subjects in the per-protocol population with no missing values during the trial. The Per-Protocol Population was chosen as it constitutes the most protocol compliant dataset, including treatment/placebo compliance, completion of planned visits including pain questionnaires and scheduled X-rays, and absence of protocol deviations such as use of analgesic treatment or surgical procedures which may interfere with pain questionnaires. A single target knee was selected for each patient, and the patient was made aware of which knee was the target knee. Target knee designation was made based on eligibility criteria as described above. If both knees were deemed eligible, the knee with the lowest eligible KL-score was selected (KL 2), and if both knees were KL 2, the knee with the highest pain score was selected as the target knee. In Study 2, data on pain in the non-target knee was only collected at baseline. For analysis of pain change of the non-target knee in subjects receiving placebo, a sub-group consisting of non-target knees matching the pain inclusion criteria as mentioned above, were selected, to improve comparability between the two groups of knees and reduce noise originating from small numerical changes in knees with very little baseline pain leading to large proportional (percentage) change. In addition, only non-target knees with no missing pain data throughout the trial were selected. Thus, 771 target knees and 256 non-target knees are analyzed.

For the analysis of baseline pain categories and risk of Joint-Space Narrowing (JSN), data of all baseline 771 target knees and contralateral 733 non-target knees with both baseline and Year 2 radiographic assessments were analyzed, regardless of baseline pain level. The studies were conducted in full compliance with the Helsinki Declaration, and were approved by all applicable Instiutional Review Boards, Ethical Committees and Competent Authorities in the countries in which they were conducted.

### Radiographic evaluation

X-ray images of both knees using fixed-flexion were obtained at the screening to assess the eligibility for study participation and to select the target knee. Each X-ray image was read by one expert radiologist for absolute JSW and KL-grade. Additional X-ray images were obtained at months 12 and 24 to, upon final data analysis, assess JSN defined as absolute change in JSW in millimeters during the 24 month period.

### Pain assessment

Pain, stiffness and function of both the target- and non-target knees were assessed by the WOMAC version VA3.1 questionnaire. In Study 2, pain in the non-target knee was only assessed at baseline. For the present analysis, only the pain sub-scale is used. The subjects were instructed to read each question carefully and mark an X on a 100 mm line, on which 0 mm equaled “No Pain” and 100 equaled “Worst Pain Imaginable”.

The pain sub-scale records patient assessments in the following five situations: 1; during walking on a flat surface, 2; using stairs (up or down), 3; at night while in bed, 4; sitting or lying, and 5; while standing. Composites of the WOMAC pain subscale were constructed to detect associations with radiographic progression: A) pain experienced while the joint was under load, and/or the patient was active (questions 1, 2, and 5) or B) pain experienced while idle and while the joint was free of mechanical load (questions 3 and 4).

### Statistical analyses

The baseline total WOMAC pain score and each pain sub-scale score was normalized to a percentage of the maximum possible score and mean levels were compared in a mixed model with pain level as the dependent variable, the question as a fixed effect, and the patient as a random effect.

The change in pain level from baseline of each patient at a given time point was calculated as a proportionate change in percent, e.g. a change from 40 mm to 20 mm equals a 50% reduction, and the mean change in percent for each variable was used. Changes in percent from baseline pain were calculated at months 1, 6, 12 and 24, and differences between individual questions were assessed using a repeated measures ANOVA. Pain values below 10 of 100 were set arbitrarily at 10 to normalize output and reduce noise. Pain by category (“weight-bearing”; WOMAC questions 1, 2 and 5 and “non-weight-bearing”; WOMAC questions 3 and 4) was similarly analyzed for change over time, and compared in a repeated measures ANOVA. Similar to the individual WOMAC scores, if at any timepoint the score of a category was below 10, the value was set at 10 to normalize output and reduce noise. Selected patient baseline characteristics; pain by WOMAC question, Joint-space width, age, sex, and body mass index (BMI) were assessed for association with PPR defined as change in percent from baseline to year two using Spearman’s correlation.

Spearman’s correlation analysis was used to assess the relationship between baseline WOMAC pain levels (levels of WOMAC pain score, each pain sub-scale question, and composite pain questions) and the radiographic progression measured as change from baseline in JSW at year two, i.e. Joint Space Narrowing (JSN), for both target- and non-target knees. Data were calculated both unadjusted and adjusted for BMI and KL-grade as predictors. These parameters have previously been shown to be associated with progression in the study population used for this analysis [22]. Results of the spearman’s correlation analyses were considered exploratory, and were not adjusted for multiplicity. To graphically depict associations between baseline WOMAC pain level and radiographic progression, all available Target (*N* = 771) and Non-Target knees (*N* = 733) of the Per Protocol population were divided by pain quintiles (A-E) for each WOMAC question (Q1–5), and plotted against JSN at year two.

### Regression to the mean

As described by Barnett and colleagues [23] the impact of regression to the mean (RTM) in the current report was estimated using a SAS-script as supplied by Barnett incorporating within- and between-patient variance in pain reporting. The estimated RTM was calculated as change in pain from baseline to year two, and then subtracted from the observed change in pain from baseline for each pain category to estimate the change in pain excluding the influence of RTM.

## Results

The baseline characteristics and mean pain levels are shown in Table 1.

**Table 1:** Demographic characteristics by study and overall per protocol population

Demographic characteristics placebo group, per protocol population				
Parameter	Target knee *N* = 771		Non-target knee *N* = 256	
Sex - *n* (%)				
Male	280 (36.3)		82 (32.0)	
Female	491 (63.7)		174 (68.0)	
Age (years)				
Mean (SD)	64.5 (6.5)		64.70 (6.3)	
Median (min, max)	64.1 (50, 81)		64.5 (50.4, 79.5)	
Age group (years) – *n* (%)				
< 65	456 (59.1)		137 (53.5)	
≥ 65	315 (40.9)		119 (46.5)	
Race – *n* (%)				
Caucasian	666 (86.4)		228 (89.1)	
Asian	103 (13.4)		27 (10.5)	
Other	2 (0.3)		1 (0.4)	
BMI (kg/m^2^)				
Mean (SD)	28.6 (4.7)		28.6 (4.4)	
Median (min, max)	27.9 (17.3, 45.9)		28.1 (19.6, 43.3)	
KL Score - *n* (%)				
KL 0	N/A		5 (2)	
KL 1	N/A		37 (14.5)	
KL 2	641 (83.1)		130 (50.8)	
KL 3	130 (16.9)		73 (28.5)	
KL4	N/A		10 (3.9)	
JSW (mm)				
Mean (SD)	3.43 (0.99)		3.15 (1.56)	
Median (min, max)	3.4 (1.8, 6.7)		3.39 (0.0, 7.3)	
WOMAC pain score (normalized 0–100)	Mean (SD)	Median (min, max)	Mean (SD)	Median (min, max)
Total pain (Q1–5)	49 (15)	47 (10, 100)	50 (16)	47 (30, 96)
Walking on flat surface (Q1)	47 (20)	47 (2, 100)	47 (21)	46 (2, 99)
Stairs (up or down) (Q2)	65 (18)	65 (2, 100)	65 (19)	63 (13, 100)
In bed at night (Q3)	42 (26)	41 (0, 100)	44 (24)	44 (0, 98)
Sitting or lying (Q4)	42 (22)	40 (0, 100)	43 (22)	43 (0, 100)
Standing(Q5)	49 (21)	49 (0, 100)	49 (20)	48 (3, 100)
Weight-bearing Composite (Q1, 2, 5)	53 (15)	52 (2, 100)	53 (17)	51 (18, 96)
Non-weight-bearing Composite (Q3, Q4)	42 (21)	40 (0, 100)	44 (21)	41 (0–96)

Trial 1: NCT00486434. Trial 2: NCT00704847. Non-target knees is a sub-group matching the pain inclusion criteria of the target knee within the per-protocol population. Both trials 1 and 2 were phase three randomized trials with a duration of 2 years, evaluating the effect of oral salmon calcitonin in symptomatic knee osteoarthritis on radiographic progression and symptoms. *BMI* Body Mass Index, *KL-score* Kellgren-Lawrence, *JSW* Joint Space Width, *WOMAC* Western Ontario and McMaster Universities Arthritis Index

### Pain categories and pain placebo reduction

A reduction in pain in patients receiving placebo was evident from month 1, at which point the mean reduction in pain from baseline was 8.1%.

WOMAC Q2 was associated with the highest reduction in pain for both Target (T), and NT (Non-Target) knees (36.8% ±52.4 (SD) and 28.0% ±44.4, respectively) at year two. Notably the observed pain reduction was higher in the T knee, compared to the NT knee at all corresponding time-points.

**Fig. 1:**
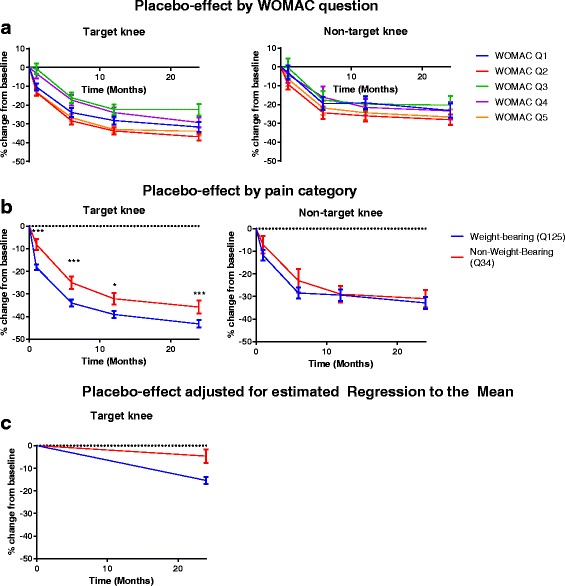
Target knee (*n* = 771) and Non-target knee (*n* = 256) mean pain change from baseline in % over 24 months, normalized (0–100), by pain question (**a**) and category; Weight-bearing pain (WOMAC questions 1, 2 and 5), and non-weight-bearing pain (WOMAC questions 3 and 4) (**b**). Baseline values below 10 are set to 10 to reduce noise. Non-target knees were selected to include only those meeting the pain inclusion criteria of the target knee for comparability. **c** depicts mean change from baseline in %, adjusted for the estimated regression to the mean as listed in Table 2. Error bars are SEM. *: *p* < 0.05 ***: *p* < 0.001

The level of placebo pain reduction was significantly higher in questions related to weight-bearing pain compared to non-weight-bearing pain. This was statistically significant in the T knee (*p* < 0.005) at month 1 and onwards, but did not reach statistical significance for the NT knee (Fig. 1). No differences were observed between questions within the two categories (weight-bearing and non-weight-bearing) at any time-points. Notably the main divergence between the two categories occurred during the first month, at which point the difference in change from baseline between the two categories is 10%; a difference which remains largely unchanged through the remainder of the trial.

Regression to the mean as a result of chance should be considered as a confounder in all assessments of change in pain over time [24]. The assessment of RTM in the current dataset (Table 2) indicates that data of pain change during the trial of the target knee may have affected by RTM for both composites and individual questions of weight-bearing and non-weight-bearing pain. When adjusting the observed pain change during the trial for the RTM estimate, the non-weight-bearing pain composite appears to change much less from baseline levels during the trial, while a modest decline in weight-bearing pain is observed (Fig. 1c).

**Table 2:** Estimated Regression-to-the-mean

WOMAC sub-score	SD	σ^2^_b_	σ^2^_w_	Estimated RTM effect from BL to Y2 (%)	Pain change from BL to Y2 adjusted for estimated RTM effect (%)
Question 1: Walking on flat surface	56	1602.5	1533.5	32.6	0.9
Question 2: On stairs	38	735.0	709.0	26.0	−10.9
Question 3: Lying in bed	80	3872.0	2528.0	33.7	11.2
Question 4: Sitting or lying	69	2551.9	2209.1	35.6	6.3
Question 5: Standing	57	1614.8	1634.3	33.9	−0.1
Questions 1, 2, and 5: Weight-bearing	39	734.6	786.4	27.8	−15,4
Questions 3 and 4: Non-weight-bearing	65	2433.6	1791.4	31.2	−4.6

The percentage of RTM of the Target knee as estimated using the method of Barnett et al. [24]. Calculation of σ^2^_w_ was based on within patient data from screening visit and subsequent baseline visit, between 1 and 3 weeks apart. σ^2^_b_: Between patient-variance. σ^2^_w_: Within patient-variance. *BL* Baseline. *Y2* Year 2, *RTM* Regression to the mean

### Baseline clinical and radiographic features and pain change in patients receiving placebo

Baseline BMI, age, sex and JSW were not associated with pain change in the placebo-treated patients at year two.

### Associations of baseline pain and radiographic joint-space narrowing

#### Target knee

In target knees, Spearman’s correlation of individual WOMAC pain questions showed very weak correlations between baseline pain score and change in JSW at year 2. WOMAC question 2 (pain while walking on stairs) was found to be negatively correlated with change in JSW (rho = − 0.079, *p* = 0.028). Consequently neither of the composite scores of pain while under load (Q1, Q2 and Q5) or pain while idle (Q3 and Q4) were significantly correlated with change in JSW at year 2. Adjustments for KL-score and BMI did not lead to significant changes in correlations between pain severity and radiographic progression.

**Fig. 2:**
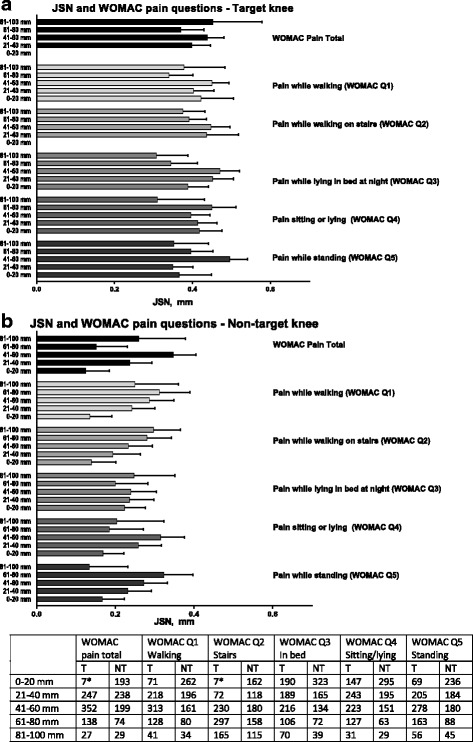
Joint-space narrowing (JSN) at year 2 by baseline pain severity level of individual and total WOMAC pain scores of target (**a**)- and non-target (**b**) knees. Data on JSN are mixed-model least-square means (standard error) adjusted for KL-score, BMI, study code, and sex. Pain severity is divided into quintile subgroups of normalized maximum possible score ranging from 0 mm (least possible pain) to 100 mm (worst imaginable pain for each WOMAC pain category. The number of observations (knees) in each category are shown in the table below the figures. Sub-groups with less than 10 observations, marked with * in the table, are excluded from the figure. Target knee. NT: Non-target knee. WOMAC: Western Ontario and McMaster’s Universities Arthritis Index. Error bars are SEM

The average JSN in target knees at year 2 ranged from 0.34 mm to 0.49 mm with little apparent influence of degree or, type of baseline pain (Fig. 2).

#### Non-target knee

As shown in Table 3, all WOMAC questions apart from Q3 (pain at night while in bed) were very weakly, positively associated with a change in JSW at year 2, indicating that a higher baseline pain score may contribute, albeit very modestly to a higher risk of progression in the NT knee. WOMAC question 5 (pain while standing), appeared most associated with progression (rho = 0.10, *p* = 0.007). The correlation coefficients of the composite score of weight-bearing pain (Q1, Q2 and Q5) to JSN were rho = 0.10, *p* = 0.007, while non-weight-bearing pain (Q3 and Q4) was weaker, with a rho = 0.05, *p* = 0.14.

**Table 3:** Correlations between baseline WOMAC scores, composite WOMAC scores and change in joint-space width (JSW) at year two

Variables		WOMAC Q1		WOMAC Q2		WOMAC Q3		WOMAC Q4		WOMAC Q5		WOMAC Weight-bearing		WOMAC idle		Total WOMAC pain score	
		adj	unadj	adj	unadj	adj	unadj	adj	unadj	adj	unadj	adj	unadj	adj	unadj	adj	unadj
Target chg in JSW yr. 2	Spearman	−0.03	− 0.01	**− 0.10**	**−0.08**	− 0.05	−0.03	0.00	0.00	0.04	0.05	−0.04	−0.02	− 0.02	−0.02	− 0.04	−0.02
	*P*	0.40	0.70	**0.006**	**0.03**	0.27	0.36	0.98	0.90	0.29	0.15	0.31	0.59	0.54	0.62	0.33	0.60
	N	771	771	**771**	**771**	771	771	771	771	771	771	771	771	771	771	771	771
Non Target chg in JSW yr. 2	Spearman	0.07	**0.09**	0.06	**0.09**	0.01	0.03	0.06	**0.07**	**0.08**	**0.10**	**0.08**	**0.10**	0.04	0.05	**0.07**	**0.09**
	*P*	0.07	**0.02**	0.09	**0.02**	0.71	0.40	0.10	**0.05**	**0.02**	**0.007**	**0.03**	**0.007**	0.27	0.14	**0.05**	**0.01**
	N	733	**733**	733	**733**	733	733	733	**742**	**733**	**742**	**733**	**739**	733	733	**733**	**733**

“Unadj” refers to unadjusted data. “Adj” is adjusted for Kellgren-Lawrence grade and Body Mass Index. Correlations with a *p*-value < 0.05 are highlighted in bold. *WOMAC* Western Ontario and McMaster’s Universities Arthritis Index

After adjustment for KL-score and BMI, WOMAC Q5 and the composite score of weight-bearing pain were rho = 0.08, *p* = 0.02 and rho = 0.08, *p* = 0.03, respectively).

JSN in the non-target knees varied from very low to moderate, as progression rates ranged from 0.13 mm to 0.32 mm during the two-year study period. Visual inspection of Fig. 2 indicates a dose-dependent association between weight-bearing pain components, particularly pain while walking on stairs and JSN, but not non-weight-bearing pain.

## Discussion

### Placebo-response

This report is the first to describe differences in pain reduction between separate pain categories in in patients receiving placebo in OA trials. Several reports have investigated biochemical, psychological and psycho-social determinants of analgesic placebo-response, but to the knowledge of the authors, no investigations regarding disease-specific categories have been performed, identifying differences in susceptibility to placebo pain reduction.

The common change in pain observed in the placebo-groups of OA clinical trials can be regarded as the sum of three distinct elements; natural OA pain progression (discussed below), regression to the mean, and the true placebo response. Using a crude estimate of RTM, we report that regression to the mean may account for a part of the observed change in particularly non-weight-bearing pain, indicating a less pronounced true placebo-response on this parameter, as opposed to weight-bearing pain. The results indicate that the impact of RTM may have been overlooked as a significant driver of change in pain over time in clinical trials, but further research is needed to draw conclusions on this issue.

The findings that measures of weight-bearing pain appear more susceptible to change in patients receiving placebo than measures of non-weight bearing pain can be interpreted in several ways: 1): These measures are simply more susceptible to placebo-response, and should therefore be limited as a means of quantifying pain response in clinical trials. 2): The weight-bearing pain-measures are more sensitive to change, and therefore also more sensitive to neurobiological changes caused psychological factors such as expectations to treatment efficacy etc., but perhaps also more sensitive to change using an efficacious treatment. 3): The non-weight-bearing pain measures may be driven by a distinct pain mechanism, perhaps central sensitization, which may be less sensitive to change in patients receiving placebo, but perhaps also less sensitive to otherwise efficacious, non-neurologically-targeted analgesic treatments. As discussed below, the current report lacks suitable controls to adequately address these questions, which will need to be addressed in experiments designed fully or in part with that purpose.

The results are interesting since they could indicate distinct differences in etiology of each pain category, as mentioned above. As can be imagined, pain experienced while the joint is free of mechanical load, such as lying in bed at night (WOMAC pain question 3), is likely to have a significantly different etiology compared to pain evoked by mechanical loading and movement. The work of Arendt-Nielsen and colleagues describes that chronic, nociceptive pain can lead to lowered pain thresholds in parts of the body remote from that of the diseased joint [5, 25]. With reference to the findings of Hensor et al., documenting the sequential emergence of first weight-bearing pain symptoms, and finally non-weight-bearing symptoms in the gradual development of OA [19], it is plausible that non-weight bearing pain itself may partly be a product of continuous, constant nociceptive pain signaling leading to central allodynia and hyperalgesia, but in itself be a poorer reflection of disease activity than weight-bearing pain. This may also help to explain some weak associations between radiographic progression of disease and pain as described in the literature [8, 26, 27].

The trajectories of pain change during the trial of both the target and the non-target knees were similar. While the differences between weight-bearing and non-weight-bearing pain was not found to be significantly different for the non-target knee, the analysis of each individual pain question revealed the same hierarchy for both knees. The overall level of change was notably higher in the target knees compared to the non-target knees, which could indicate a contribution to the placebo-effect derived from increased patient focus on the knee under study.

Intuitively, removal of the source of pain should lead to elimination of pain, but studies of total joint replacements, which indeed removes the primary source of pain, are discrepant. In trials evaluating the outcome of total knee arthroplasty, particularly on pain, results show either complete elimination of pain [28] or that while a majority of patients experience a significant reduction in pain, pain does not seem to be completely abolished [25, 29], and it has been reported that approximately 15% of patients continue to experience severe pain even after joint replacement surgery [30]. The lack of complete pain resolution might indicate a residual, non-joint specific pain mechanism, such as a state of central sensitization persisting even after surgical removal of the stimuli initiating this condition, which could render the patient less likely to respond to any analgesic treatment without undergoing arthroplastic surgery. The relevance of this observation is the presence of a possibly less dynamic pain category, which is harder to manipulate and may have a central origin rather than a peripheral. As the current results indicate that the elements of non-weight-bearing pain are less likely to change by placebo-treatment, this could be in support on this hypothesis, yet more research is needed.

The main limitations of this report is the lack of an untreated OA control, as well as a positive control. Inclusion of an untreated OA control would facilitate comparisons to a natural development of OA pain over time to determine the true placebo-response. In an analysis of pain trajectories in patients with established OA from the Cohort Hip and Cohort Knee study (CHECK), it was found that only 3% of the sample size experienced a major regression in pain, while 67.7% of subjects experienced constant or worsening pain during the 5 year follow up [31]. While it is common that OA placebo-treatments lead to placebo-response [12], the majority of OA patients participating in trials as untreated controls are not expected to achieve a notable regression of pain, nor a significant increase in pain over time [32–34]. It is therefore regarded as plausible that the observed reduction in pain in the current analysis is to a large extent attributable to a true placebo-response and potential effect of RTM rather than a result of natural history. The inclusion of an untreated control group would facilitate analyses of sensitivity to change in each pain category, as well as an estimate of the impact of the patient-practitioner interaction without medical treatment per se. This limitation is important, as the implications of the findings are not necessarily to exclude patients with a high level of weight-bearing pain, but perhaps rather to expand the view on OA pain: From one single concept to at least two possibly distinct elements of disease, which may require hitting multiple targets to be affected.

The use of subscore and individual WOMAC items to category pain may appear crude in relation to speculation regarding associations with the potential underlying mechanism of pain; neuropathic, central sensitization etcetera, particularly in the presence of other, specialized psychometric tools and devices developed to assess these particular pain elements [15, 16]. But in the absence of more or less cumbersome and experimental methods of quantifying separate pain features, as the most widely used, and simple method of assessing OA pain, the WOMAC questionnaire may provide a suitable basis for hypothesis generation to be further evaluated using more specialized tools in adequately designed trials.

### Pain categories and risk of radiographic progression

**Fig. 3:**
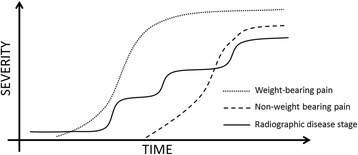
Hypothetical depiction of pain category severity and progressive disease stage over time. While disease progression is likely occurring in limited periods follow by periods of inertia [35], pain trajectories appear to be progressing more steadily, although slowly [19, 32, 33]. Weight-bearing pain is the first pain category to present, followed by non-weight-bearing pain [19]. Variable radiographic disease progression rates may blur the otherwise expected hypothetical association between pain severity and risk of progression at one or very few given time points

The weak correlations between different categories of pain and progression are in line with previous findings that pain is not clearly associated with radiographic progression [8, 35]. This supports the current belief that progression is likely associated with several factors affecting JSN, of which pain is only one. The lack of clarity between findings of the NT and the T knee, and the very weak correlations limits the options for clinical translation of the results. As a number of different readers were engaged in the radiographic readings and there was no assessment of inter-reader reliability, the resulting measurement error may contribute to the low correlations reported in Table 3. The results do indicate differences, although of unknown clinical significance, between elements of weight-bearing, and non-weight-bearing pain, most clearly shown for questions of weight-bearing pain (WOMAC questions 1,2 and 5) in the NT knee in Fig. 2. These findings warrant further research. The reasons for the discrepancies between the observations of pain and progression in the T- and NT knees are unknown. As described by Felson and colleagues, OA disease progression is likely to be characterized as a state of inertia, followed by periods of progression [36], while Hensor as well as the data in this report indicates that pain progression may evolve at a more constant rate, although weight-bearing pain surfacing before non-weight-bearing pain [19]. This discrepancy in progression rate between objectively measurable disease parameters and pain may limit the possibilities of assessing any association between pain severity and the risk of radiographic progression at one or few given point(s) in time, while an association may be found at other times, as illustrated in Fig. 3.

Due to inclusion criteria, the distribution of pain in the target knees is concentrated in the range of moderate pain. This is likely to have affected the correlation between pain and progression of the target knee, which may introduce a bias towards a lower correlation coefficient as low pain values with low progression rates are not present or very scarce.

## Conclusions

These results indicate that the reduction in pain in patients treated with placebo is different between pain categories, as weight-bearing pain was significantly more reduced compared to non-weight-bearing pain. Further research in pain categories in OA is warranted.

## Abbreviations

ANOVA: Analysis of Variance
BMI: Body Mass Index
CHECK: Cohort Hip and Cohort Knee
JSN: Joint Space Narrowing
JSW: Joint Space Width
KL: Kellgren-Lawrence
NT: Non-target
OA: Osteoarthritis
OAI: Osteoarthritis Initiative
PPR: Placebo pain reduction
RTM: Regression to the mean
T: Target
WOMAC: Western Ontario and McMasters Osteoarthritis Index
